# The Role of LRP1 in Glioma Progression and Therapeutic Targeting: A Narrative Review

**DOI:** 10.3390/cells15131163

**Published:** 2026-06-26

**Authors:** Muhanad Alhujaily

**Affiliations:** Department of Biochemistry, College of Medicine, Imam Mohammad Ibn Saud Islamic University (IMSIU), Riyadh 13317, Saudi Arabia; malhujaily@imamu.edu.sa

**Keywords:** blood–brain barrier, lipoproteins, LDL, glioma, prognosis, transcytosis

## Abstract

Gliomas are the most frequently encountered tumors in the central nervous system, with limited therapeutic effectiveness owing to their highly invasive nature, intratumoral heterogeneity, and presence of the blood–brain barrier (BBB). Low-Density Lipoprotein Receptor-Related Protein 1 (LRP1) is a large, multifunctional transmembrane endocytic receptor that regulates lipid metabolism, cell signaling, and endocytosis in various body tissues, including the brain. LRP1 mediates tumor cell proliferation, invasion, and angiogenesis in gliomas through various cellular signaling mechanisms, including the SP1/PI3K/AKT pathway and MAPK/ERK. The occurrence of LRP1 in the BBB and the recent identification of its increased expression in gliomas have suggested it as a promising therapeutic target for receptor-mediated nanoparticle delivery and treatment of gliomas. LRP1-mediated transcytosis is now being used to enhance the BBB penetration of chemotherapy drugs and radiosensitizers in gliomas, which has resulted in increased overall survival of patients secondary to increased antitumor effectiveness of therapies. Despite the effective preclinical role of LRP1-targeted therapy in glioma models, clinical translation is challenging due to significant heterogeneity in the expression patterns of LRP1 across various subtypes of gliomas, which may affect the clinical responsiveness of drug therapy. Furthermore, concerns related to the pharmacokinetics of therapy and receptor saturation kinetics have rendered its clinical applicability challenging.

## 1. Introduction

Gliomas are the most frequently encountered tumors in the central nervous system (CNS), accounting for almost 81% of primary CNS tumors, with glioblastoma (GBM) being the most aggressive subtype, associated with poor prognosis [1]. Despite advancements in neurosurgical and oncological care, the median survival of GBM ranges from 12 to 15 months [2]. Therapeutic effectiveness in gliomas is basically limited owing to its highly invasive nature, intratumoral heterogeneity, and the presence of the blood–brain barrier (BBB), leading to increased risk of recurrence [3]. Hence, identification of tumor biology in terms of the molecular mechanisms that underlie its pathological cycle is necessary to offer potential avenues for therapeutic intervention.

Recently, with the advancement in knowledge regarding receptor-mediated signaling pathways involved in the progression of gliomas and their metabolic reprogramming, Low-Density Lipoprotein Receptor-Related Protein 1 (LRP1) has gained attention as an essential regulator of tumor biology in gliomas [4]. LRP1 is a large, multifunctional transmembrane endocytic receptor belonging to the low-density lipoprotein receptor (LDLR) family. LRP1 modulates multiple biological processes through its interaction with extracellular ligands and intracellular proteins, hence regulating cell proliferation and survival. It is widely expressed in various body tissues, including the brain, where it regulates lipid metabolism, cell signaling, and endocytosis [5].

Recent studies have revealed increased expression of LRP1 in GBM when compared with low-grade gliomas or normal brain tissue [6]. This increased expression leads to tumor progression via enhanced cell proliferation, increased tumor cell survival, and migration [6]. LRP1 regulates the invasiveness of tumor cells via modulation of oncogenic cellular signaling pathways like PI3K/Akt and MAPK signaling, as well as extracellular degradation of matrix metalloproteinase (MMP) [7,8].

Apart from regulation of intracellular signaling, LRP1 is known to mediate lipid and cholesterol metabolism in the brain. Unlike other body tissues, the BBB in the brain is impermeable to cholesterol, and the transport of cholesterol is mediated here by transport mechanisms, including LRP1 [9]. In gliomas, the rapid proliferation and survival of cells largely depend upon cholesterol uptake by tumor cells mediated by LRP1 [4]. Any disruption in the function of LRP1 results in reduced uptake of cholesterol by tumor cells, hence affecting tumor cell survival [4]. Additionally, LRP1 plays an essential role in regulating the tumor microenvironment and mediating host–tumor cell interaction [10]. The increased expression of LRP1 in highly angiogenic areas of the tumor reflects its role in angiogenesis [11]. During hypoxic conditions, LRP1 is known to promote motility and invasion of GBM cells via its interaction with cellular signaling mechanisms, including the PI3K/Akt pathway [12].

These multifunctional abilities of LRP1 have therefore rendered it a potential therapeutic target in gliomas. Unfortunately, the literature regarding the role of LRP1 in gliomas is deficient in large-scale clinical studies and is limited only to experimental models with large heterogeneity. This necessitates the need for comprehensive evidence to determine LRP1’s multifaceted role in tumor cell biology, its role as a therapeutic target, and a biomarker in gliomas.

This narrative review provides comprehensive evidence of the structure of LRP1, the mechanisms involved in the biology of glioma cells, as well as its therapeutic implications.

## 2. Methodology

A narrative literature review was conducted to determine the role of LRP1 in the biology of glioma cells as well as its therapeutic implications using PubMed and Google Scholar as databases. We included all the relevant original articles, experimental studies, translational research, and review articles published from inception to April 2026 using multiple keywords including “LRP1”, “Gliomas”, “Blood-Brain barrier”, “tumor microenvironment”, “nanoparticles”, “glioma biomarkers,” and “receptor-mediated transcytosis”.

All articles that were published in a language other than English, conference abstracts, and articles not providing direct relevance to gliomas were excluded.

## 3. LRP1—Structure and Biology

**Figure 1:**
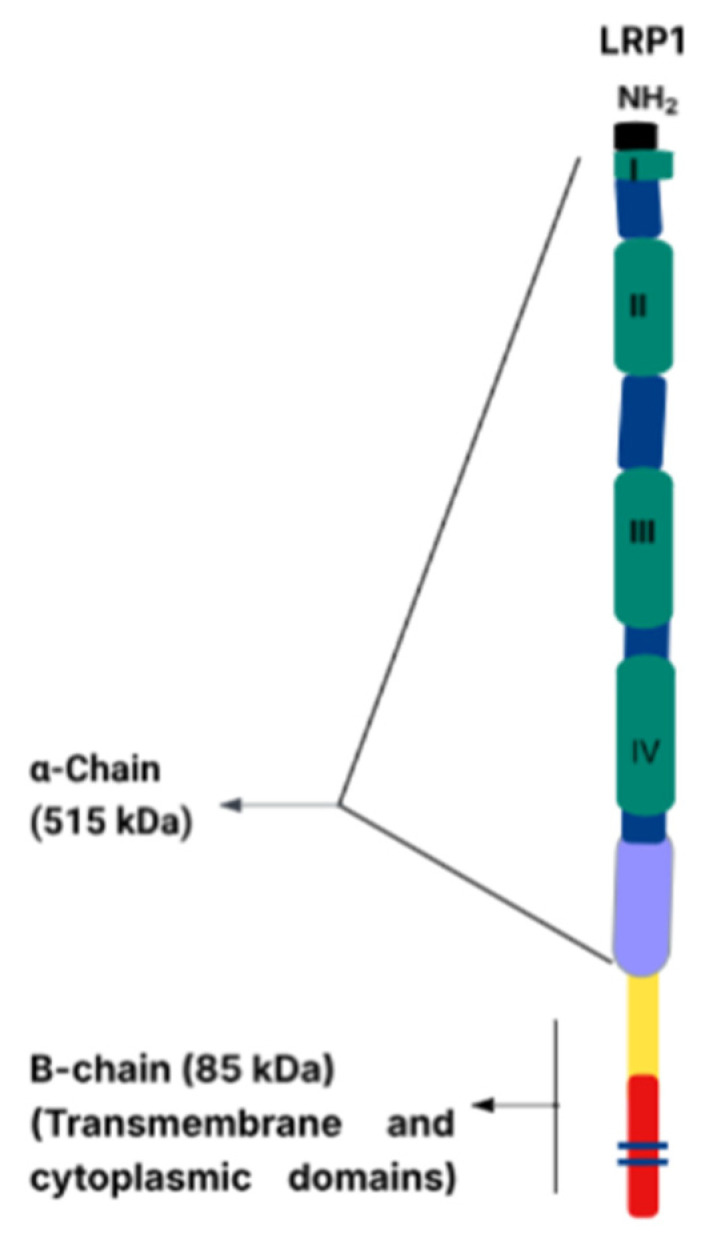
Structure of LRP1.

LRP1, Low-Density Lipoprotein Receptor-Related Protein 1, is the largest multifunctional scavenger, signaling receptor, and transporter belonging to the LDL receptor family with an approximate weight of almost 600 kDa [13,14]. After its synthesis as a polypeptide chain via transcription from the LRP1 gene on chromosome 12q13-q14, LRP1 is cleaved by furin in the trans-Golgi complex into an alpha- and a beta-chain. The alpha-chain weighs approximately 515 kDa with four ligand-binding domains, including two major ligand-binding domains, i.e., Cluster II and Cluster IV, which bind to more than 50 ligands with great structural diversity, and Cluster I and Cluster III. These clusters are made up of cysteine-rich complement-type repeats (CCRs), including 2, 8, 10, and 11 CCRs [9,15,16]. These clusters are separated from each other via epidermal growth factor precursor repeats (EGFrs) comprising two cysteine-rich EGFrs, one YWTD (beta-propeller domain) repeat, followed by another EGF-like repeat [5]. Conversely, the cytoplasmic beta-chain is comparatively lighter with a weight of approximately 85 kDa. It comprises a transmembrane domain connected to the alpha-chain and a cytoplasmic domain, which consists of two NPxY motifs. The LRP1 is further cleaved by β-Secretase to soluble LRP1 after cleavage of the extracellular alpha-chain from the transmembrane domain of the beta-chain, leaving behind the C-terminal fragment of LRP1 (LRP1-CTP) [17,18]. The structure and cleavage of LRP1 are provided in Figure 1.

## 4. LRP1 in Gliomas

Glioma cell survival is largely dependent upon cholesterol metabolism, yet the levels of cholesterol-producing enzymes in these cells are deficient, which explains the transport of cholesterol from the surroundings. Cholesterol is necessary for tumor cell proliferation owing to its essential role in cell membrane synthesis. In cancers cell, there is aberrant uptake of LDL through the clathrin-mediated pathway to balance the higher proliferation of cancer cells [19]. In the brain, the uptake of LDL is limited by the BBB, and owing to downregulation of intracellular cholesterol-producing enzymes, the receptor-mediated transcytosis of LDL is also limited. An increased concentration of 1-oleyl cholesterol and tetrahydrocorticosterone in the cerebrospinal fluid and an increased serum concentration of LDL in grade III glioma patients support their role in malignancy [20]. Maletinska et al. reported an increased expression of LDL receptors, especially LRP1, in several cell lines of glioma models, including SF-767, SF-763, A-172, U-87 MG, U-251 MG, U-343 MG, and SF-539 [21].

### 4.1. Role of LRP1 in Glioma Cell Migration and Invasion

Kuang et al. described the role of LRP1 in the migration and invasion of GBM via epithelial–mesenchymal transition (EMT) and the SP1/PI3K/AKT pathway [22]. LRP1-mediated blockade of EMT results in a rise in levels of E-cadherin and increases in vimentin levels, evident on Western blot analysis. Additionally, decreased expression of SP1 and phosphorylation of AKT after knockout of LRP1 show that LRP1 may promote migration of gliomas via SP1/PI3K/AKT signaling cascade [22]. Mast cell chemotaxis to develop a tumor microenvironment stimulated by the secretion of PAI-1 through glioma cells is also mediated via LRP1 receptors. The LAD2 cells and mast cells in glioma cells express LRP1. The interaction of PAI-1 with LRP1 results in increased mast cell migration towards glioma cell media via activation of the STAT3 pathway, followed by degranulation and release of inflammatory mediators such as histamine, TNF-α, and several others, resulting in tumor invasion, angiogenesis, and modulation of the tumor microenvironment [23].

### 4.2. Role of LRP1 in Glioma Cell Proliferation and Interaction with Tumor Microenvironment

LRP1 results in the proliferation of tumor cells via activation of the ERK pathway and inhibition of the JNK signaling cascade [7], a positive regulator effect over the AKT/NF-κb pathway [8], decreased NOTCH signaling [24], and apoptotic pathway mediation via caspase 3 [8]. LRP1 regulates the proliferative activity of glioma cells via increased expression of MMP2 and MMP9 [25]. Almost 90% of LRP1 silencing results in a 50–60% reduction in expression levels of MMP2 and MMP9, with a great effect on MMP2 [25].

Tumor microenvironment (TME) comprises all the molecular and cellular components that facilitate the progression of the tumor. In gliomas, apart from tumor cells, some non-cancer cells, including pericytes, endothelial cells, fibroblasts, and tumor-associated macrophages (TAMs), comprise almost 40% of the TME as immune cells [26,27,28]. The interaction of tumor cells with TAMs is essential for the progression of tumor growth via enhanced angiogenesis, stimulation of cancer stem cells, and promotion of an immunosuppressive TME [29].

In gliomas, M2 subtype macrophages play an important role in the progression of tumor activity via promotion of tumor invasion, migration, and intravasation. Additionally, it inhibits the antitumor activity of natural killer cells and T cells, a major hallmark for poor response to chemotherapy [29]. LRP1 affects the TME indirectly via MDK, a protein expressed by EGFRvIII-mutant GBM, which mediates tumor angiogenesis and growth [30]. Kuang et al., while developing a machine learning model for the prediction of prognosis of gliomas, proposed LRP1 as a significant prognostic gene along with other lactylation-associated genes, LOX1 and APOD [22]. The increased IGF-1 activation in glioma cells with increased lactylation scores shows that the proliferation and migration of gliomas is mediated by IGF-driven signaling pathways, including MAPK/ERK signaling and the PI3K/AKT pathway, which indicate their role as prognostic biomarkers of glioma [22].

### 4.3. Role of LRP1 in Glioma Cell Angiogenesis

Apart from immunohistochemical staining in tumor cells, an increased expression of LRP1 is also observed in vascular endothelial cells of glioma tissues, which might depict their essential role in neovascularization and angiogenesis [6]. Song et al. have also postulated the possible role of LRP1 in neovascularization via its positive correlation with urokinase-type plasminogen activator receptor expression, which mediates migration of endothelial cells and matrix degradation [25].

**Figure 2:**
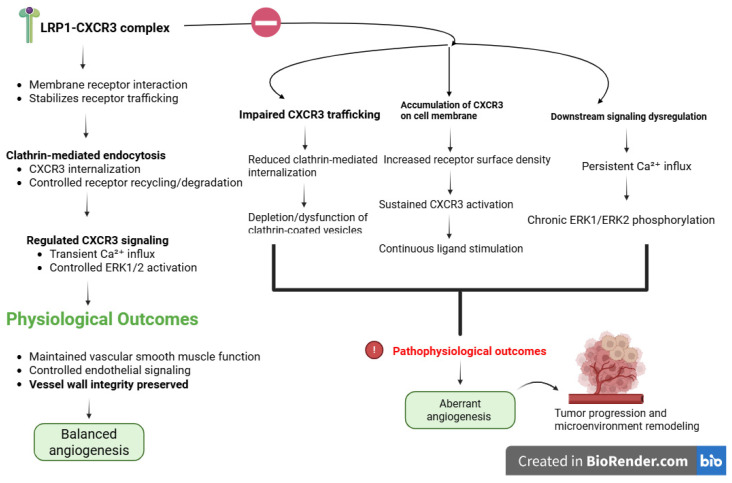
LRP1-CRC3 complex in glioma cell angiogenesis. Created in BioRender. Alhujaily, M. (2026). https://app.biorender.com/illustrations/6a0f1d5f04096164d521c211?slideId=32d841b8-6300-41d6-aca7-1be4dd52a627 (accessed on 15 June 2026).

LRP1 regulates angiogenesis owing to its ability to maintain the integrity of the vessel wall via PDGFRβ-dependent activation of PI3K [31]. It leads to invasion of tumor cells as well as neovascularization, resulting in tumor metastasis and progression [32]. LRP1-silenced cells typically have a decreased level of vascular endothelial growth factor, which describes their significance in angiogenesis [32]. LRP1 mediates the response of endothelial and megakaryocyte cells to chemokine receptors (CXCs) and CXCL4 chemokine [33,34]. It modulates the activity of GPCR S1P (Gi-dependent sphingosine-1-phosphate) signaling by inhibiting the Gαi subunit without interaction with S1P receptors [35]. It plays an essential role in the activation of CXCR3 and its trafficking. The CXCR3-LRP1 complex, present in both tumor cells and smooth muscle cells, results in the maintenance of the integrity of the vessel wall [36]. The internalization of CXCR3 is largely dependent upon clathrin-mediated transport via LRP1 downstream. Loss of LRP1 may result in depletion of clathrin-coated vesicles, decreased internalization of CXCR3, increased CXCR3 at the cell membrane, continuous stimulation, and sustained increase in calcium efflux through the cell membrane and constant stimulation of ER1/ERK2 phosphorylation [36] (Figure 2).

## 5. Molecular Heterogeneity of LRP1 Expression

The substantial heterogeneity of gliomas at both the cellular and molecular levels affects the clinical outcomes of the disease via altered tumor progression and therapeutic responsiveness. As for all other receptor-mediated signaling pathways involved in the biology of gliomas, the expression of LRP1 across various gliomas, depending upon their histopathological grading, molecular subtypes, and heterogeneous tumor microenvironment, is quite variable, which may limit the effectiveness of various therapeutic approaches [6,22,37].

Recent molecular analysis of glioma cell lines by Shrithi et al. revealed a significant variation in expression patterns of LRP1 mRNA across low-grade and high-grade glioma cells, with the highest expression in GBM cells, followed by anaplastic astrocytoma and low-grade astrocytomas, i.e., 87.5% to 91.7%, 50% to 61.5%, and 42.9% to 44%, respectively [6]. While incorporating the IDH-mutant status of glioma cells, the researchers observed a potential relationship between LRP1-mRNA expression and the aggressive IDH phenotype of IDH-wildtype tumors, with significantly greater expression of LRP1 in IDH-wildtype tumors when than in IDH-mutant grade 4 astrocytomas [6]. Similarly, the experimental glioma models in a study by Yang et al. also proposed a decrease in expression of LDLRS and LRP1 in IDH-mutant gliomas when compared with that in IDH-wildtype gliomas [37]. In RNA sequencing gene analysis of glioma cells, LRP1 showed greater expression in late-stage microglial cells, which are associated with poor clinical outcomes [22].

Although the exact mechanism underlying this association of IDH-associated metabolic reprogramming of glioma cells has not been fully elucidated, the regulation of LRP1 expression in glioma cells via alterations in the metabolism of cholesterol and liver X receptors (LXRs) may justify this association [37]. Increased levels of 24-hydroxycholesterol in gliomas result in activation of LXRs, which promotes the inducible degradation of the LDL receptor (IDOL)-mediated degradation of LDLRs, including LRP1, to reduce receptor-mediated uptake of cholesterols. This leads to compensatory activation of sterol regulatory element-binding proteins (SREBPs) and downstream signaling cascades, which may explain the altered sensitivity of gliomas to statins [37].

Apart from molecular status, intratumoral heterogeneity in localization patterns of LRP1 is also observed in various histopathological grades of gliomas, with predominant membranous and cytoplasmic immunoreactivity of LRP1 in high-grade gliomas and localized perinuclear staining in low-grade astrocytomas. This may reflect the role of LRP1-mediated signaling in determining the aggressiveness of tumors and the need for precision targeted therapy [6].

Unlike other cancer cells, where the expression patterns of LRP1 are altered by the methylation status of tumors [38], Yang et al. observed no difference in expression levels of LDLRs, including LRP1, in glioma models when treated with DNA methylation inhibitors like decitabine [37].

Collectively, these findings indicate the dynamic expression of LRP1 across various molecular subtypes, which may have important implications for the clinical translation of LRP1-targeted therapy. The decreased LRP1 expression in IDH-mutant gliomas may limit the effectiveness of nanoparticle-based therapy in comparison to IDH-wildtype gliomas. Additionally, the intratumoral heterogeneity in receptor density may affect the penetration of drugs among patients, yet the literature needs further studies to develop reproducible evidence for clinical translation.

## 6. LRP1 Therapeutic Implications and Nanomedicine

The BBB is a significant challenge in the treatment of gliomas, as it can block almost all large molecules and 98% of small molecules from passing through it [39]. The limited clinical effectiveness of temozolamide in gliomas and its peripheral tissue damage due to extra-CNS accumulation is also attributed to the limited penetration of the drug via the BBB [40]. Receptor-mediated transcytosis (RMT) is a critical mechanism to overcome BBB penetration issues in CNS tumors. Multiple receptors that are involved in RMT across the BBB have been identified till now, including insulin-like growth factor-1 receptor (IGF1R), TfR, insulin receptor (IR), neonatal Fc receptor (FcRn), angiotensin-converting enzyme receptor (ACE), and LRP1 receptor [41]. Although all these receptors have been extensively utilized to exploit the BBB for treatment of gliomas [42], their use is limited to some receptor-related characteristics. For instance, the targeting of TfR raises concerns associated with competition with endogenous transferrin and endosomal sequestration [43]. Wang et al. described the competitive inhibition effect produced by excessive endogenous Tf (2250 Nm) over Tf-modified drug delivery systems [44]. Preston et al. described the intracellular physiological trafficking of transferrin and receptor-mediated constraints [45]. Similarly, the disassociation of ligand from transferrin receptors is largely affected by pH [46]. Additionally the broader expression of TfR in normal body tissues other than the CNS, including the liver, spleen, and placenta, reduces tumor specificity and may result in off-target uptake [47]. Georgivea et al. also discussed the limited efficacy of transferrin receptor-mediated drug delivery systems due to systemic constraints [48]. Insulin receptor-mediated targeting results may lead to physiological alterations and metabolic reprogramming [49]. The potential competition between endogenous insulin and therapeutic drug ligands for binding to receptor and receptor trafficking may disturb glucose homeostasis, body physiology and efficiency of drug delivery [48]. Contrary to this, LRP1 is potentially advantageous due to its dual expression in both BBB endothelial cells and glioma cells [41], yet the comparative literature of optimal targeting strategy and receptors for clinical translational purposes is limited and needs to be addressed in future studies.

Due to the increased expression of LRP1 in gliomas, RMT via LRP1 has now been utilized to improve treatment effectiveness in gliomas [41]. Inhibition of LRP1 has also proved to be effective in limiting the adhesion of Polymorphonuclear neutrophils (PMNs) by inhibiting the effect of MDK in gliomas [30]. A significant improvement in penetration of the BBB and protein delivery to the orthoptic GBM model has been observed for Apolipoprotein E (LRKLRKRLLLRKLRKRLLC) due to its ability to bind with LDLRs [50,51]. Wei et al. reported improved penetration, cellular uptake, and antitumor activity of mLDLR-loaded sorafenib towards U-87 MG cells, increasing tumor repression and improving survival via enhanced apoptosis and reduced angiogenesis in tumor cells [52].

In nanoparticle-based therapy, targeting of the LRP1 receptor via angiopep-2 has gained recent interest owing to its ability to cross the BBB and target gliomas. The use of nanoparticles functionalized with Apolipoprotein E has shown approximately 1.5-fold greater penetration through the BBB when compared with non-functionalized nanoparticles [53]. Increased Angio-pep2 peptide, TFFYGGSRGKRNNFKTEEY, derived from aprotinin, has been known to act as an excellent drug carrier through the BBB via LRP1-mediated receptor transcytosis. Poly(amidoamine) (PAMAM) dendrimer has been shown to improve the anti-glioma effectiveness of doxorubicin and survival of gliomas both in vivo and in vitro. Additionally, the overall survival of gliomas was 31 days for Ang2-modified dendrimer (P4PAD) when compared with free doxorubicin (19 days) [54,55]. Similarly, L-D-I/NPs designed as versatile biomimetic nanoplatforms effectively inhibit tumor proliferation and prolong their survival by crossing the BBB after binding to LRP1 and inducing ferroptosis in glioma cells [56]. The intravenous injections of Ti@FeAu-Ang (angiopep-2-decorated titanium-alloy core–shell magnetic nanoparticles) and Au-DOX@PO-ANG have also been shown to decrease tumor volume in glioma cells effectively by inducing hyperthermia and coagulative necrosis after penetration into the cell via LRP1-mediated transport [57,58]. RAP, a competitive inhibitor of the LRP1 ligand, has resulted in improved neurorecovery in stroke patients and may serve as a potential therapy in GBM [59].

In photothermal therapy, the functionalization of near-infrared (NIR)-responsive gold nanorods (GNRs) with Angi-2 resulted in enhanced cellular uptake and enhanced photothermal killing of C6 cells owing to increased production of reactive oxygen species [60]. Zhu et al. modified paclitaxel with angiopep-2-modified lipid-coated mesoporous silica nanoparticles (ANG-LP-MSN-PTX), which demonstrated 10.7% greater penetration ability in intracranial C6 glioma rats and improved survival [61].

The conjugation of gold nanoparticles and doxorubicin with angiopep-2 as Au-DOX@PO-ANG in the form of angiopep-2-conjugated pH-sensitive polymersomes resulted in increased survival time in human UG-M78 xenograft mouse models [62]. Saporin, a protein toxin for gliomas, also expressed enhanced antitumor activity with limited side effects when functionalized with angiopep-2-directed and redox-responsive virus-mimicking polymersomes [63]. Contrarily, the downregulation of LRP1 by RAP has resulted in the development of a proinflammatory environment [64].

Apart from the delivery of chemotherapeutic drugs, LRP1-mediated transcytosis has also been used to enhance the BBB penetration of radiosensitizers, including Dbait in gliomas, hence improving the responsiveness of gliomas to radiotherapy [65]. Zhang et al. developed a tumor microenvironment-based angiopep-2-modified micellar system (ch-K5(s-s)R8-An) for codelivery of Dbait (a radiosensitizer) and doxorubicin, which resulted in enhanced degrees of apoptosis and DNA damage in tumor cells, reducing the proliferation and viability of U251 cells by 16.3% and 54% respectively via LRP1-mediated transport across the BBB and improved median survival of UG251 cells from 26 to 56 days [65]. Angiopep-2-modified lipid-polymer nanoparticles of temozolamide (A2-P(MIs)25/TMZ) also ward off the risk of cell toxicity associated with temozolamide and radiotherapy and enhance encapsulation of the drug to ensure radiosensitization and chemosensitization of hypoxic cells [66]. Similarly, Lu et al. reported an increase in in vitro uptake of doxorubicin in cells and on MMT assays when given in the form of angiopep-2-conjugated PS loaded with DOX in glioma-bearing rats [67]. Liu et al. also reported enhanced therapeutic efficacy of doxorubicin conjugated with angiopep-2 and epidermal growth factor receptor (EGFR)-targeting peptide (EP-1) in gliomas owing to enhanced targeting effect of the drug in glioma cells in both in vitro and in vivo studies [53]. ACHL, an all-in-one nano-sensitizer comprising Ce6 (chlorin e6) and HCQ (hydroxychloroquine) in angiopep-2-modified liposomes, resulted in an increased antineoplastic effect of sonodynamic therapy, increasing the accumulation of the drug in glioma cells and reducing toxicity to other cells [68]. Similarly, in ferroptosis therapy for the treatment of glioma, the issues regarding poor penetration of drugs could be overcome by using angiopep-2 peptide-modified engineered exosomes in the form of magnetic nanoparticles, which trigger LRP1-mediated transcytosis followed by disintegration of dihydroorotate dehydrogenase and the glutathione peroxidase 4–ferroptosis defense axis with Fe_3_O_4_ nanoparticle-mediated Fe^2+^ release and improved targeting of glioma cells [69].

Recently, KS487, a novel cyclic peptide, has demonstrated greater LRP-binding affinity and BBB penetration in in vitro models when compared with angiopep-2, potentially owing to comparatively improved plasma stability and receptor specificity, highlighting the role of drug pharmacokinetic optimization in therapeutic efficacy [69,70], yet the literature comparing the pharmacokinetics of various LRP1-targeted therapies is lacking, limiting the identification of clinically translatable platforms. Furthermore, it should be noted that the improved BBB permeability in KS487 was associated with prolonged incubation time in vitro models but limited in vivo half-life with increased systemic clearance, which highlights a significant therapeutic challenge associated with peptide-based LRP1-targeted therapy in gliomas [70].

Despite consistent BBB penetration and antitumor efficacy proven by these preclinical studies, the clinical translation of angiopep-2-based nanoparticle therapy for gliomas is limited. In a recent clinical trial of BBB-permeable peptide–paclitaxel conjugate ANG1005, Dmello et al. demonstrated modest overall therapeutic efficacy, especially in bevacizumab-refractory gliomas, with ANG1005-associated reported adverse events in 28.8% patients [71]. Similarly, Drappatz et al. reported modest therapeutic efficacy of angiopep-2-GRN1005 in high-grade gliomas, although the intratumoral concentration of paclitaxel remained high when compared with plasma level, with prolonged plasma retention, decreased systemic clearance, and absence of immunogenicity [72]. Notably, these trials reported adverse events already known in paclitaxel therapy, which implies that the systemic toxicity associated with chemotherapy drugs is not fully eliminated despite improved CNS penetration [71,72]. Additionally, improved overall survival was noted in patients with increased expression of SSR3, a paclitaxel-susceptibility biomarker, which may imply that the associated effectiveness cannot be attributed only to improved BBB penetration; the companion diagnostic biomarkers should be considered in future clinical trials while designing precision-based LRP1-targeting therapies for gliomas [71].

Moreover, the RMT-mediated drug penetration is limited owing to issues related to receptor saturation. In a recent study by Awad et al., an increase in the concentration of ANG-2-mediated nanoformulation from 10% to 20% fails to improve the transport efficiency of the drug, which might suggest receptor saturation [73]. The problem of saturation of LRP1 by angipep2 ligand in drug delivery during glioma treatment could be overcome by conjugating liposomes with TAT. Han et al. developed DOX-TAT-Ang-LIP (doxorubicin liposomes loaded with TAT and Angiopoietin-2), which showed improved penetration of doxorubicin in glioma cells followed by their necrosis [64]. Although the process of physiochemical optimization via PEGylation has been shown to overcome this issue related to receptor saturation, the evidence regarding the use of PEGylated ANG2-functionalized zein nanoparticles is in the initial stage, with available evidence limited to experimental studies, rendering its clinical translation unclear. The summary of LRP1-based therapy and its observed roles in glioma is provided below in Table 1.

## 7. Challenges and Future Recommendations

**Figure 3:**
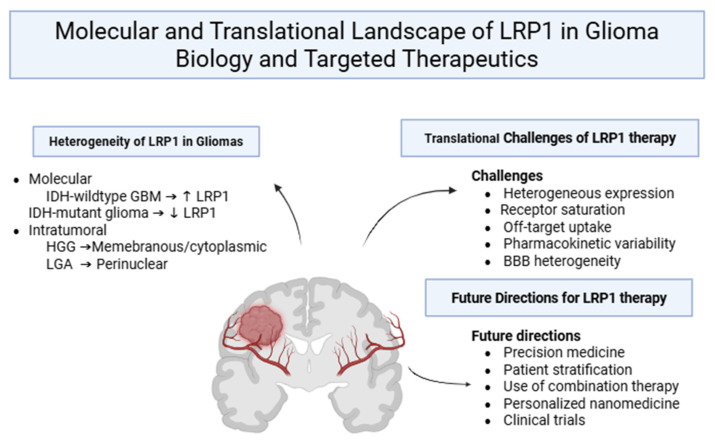
Molecular and translational landscapes of LRP1 in glioma. Created in BioRender. Alhujaily, M. (2026) https://app.biorender.com/illustrations/6a0d8e11cfc40c5f8c48d2ee?slideId=c3487a2c-36ee-4801-865f- (accessed on 15 June 2026).

Despite the promising potential therapeutic efficacy of LRP-targeted therapy in glioma management, clinical translation is hindered due to several limitations. Most of the evidence identified is based on in vitro and in vivo experimental studies with limited clinical trial validation. Additionally, the heterogeneous expression patterns of LRP1 across various histopathological grades and molecular subtypes of gliomas and intratumoral heterogeneity in LRP1 immunostaining patterns may influence the therapeutic reproducibility and effectiveness. Concerns related to the pharmacokinetics of LRP1-targeted therapy, including receptor saturation, ligand affinity, and lack of long-term follow-up profiles, may potentially affect the clinical translation of these findings, highlighting the need for large clinical studies integrating molecular stratification, spatial tumor heterogeneity analysis, and patient-specific biomarkers to establish the safety and clinical applicability of LRP1-targeted therapeutics in gliomas. A summary of the molecular and translational landscapes of LRP1 in glioma is provided in Figure 3.

## 8. Conclusions

LRP1 mediates tumor cell proliferation, invasion, and angiogenesis in gliomas via various cellular signaling mechanisms, including the SP1/PI3K/AKT pathway and MAPK/ERK. LRP1-mediated transcytosis is now being used to enhance the BBB penetration of chemotherapy drugs and radiosensitizers in gliomas, which has resulted in increased overall survival of patients secondary to increased antitumor effectiveness of therapies. Despite the effective preclinical role of LRP1-targeted therapy in glioma models, the clinical translation is challenging due to significant heterogeneity in the expression patterns of LRP1 across various subtypes of gliomas, which may affect the clinical responsiveness of drug therapy. Furthermore, concerns related to the pharmacokinetics of therapy and receptor saturation kinetics have rendered its clinical applicability challenging. The lack of human clinical trials, the variability in expression patterns of LRP1 in glioma subtypes, and the need to modulate LRP1-mediated therapy to optimize the signaling of the proinflammatory cascade are essential literature gaps to be filled in future studies.

## Figures and Tables

**Table 1:** LRP1-based therapy in glioma.

LRP1-Based Glioma Therapy Groups	Therapeutic Agent	Role in Glioma
mLDLR loaded [52]	mLDLR-loaded sorafenib	Enhanced apoptosis and reduced angiogenesis
Angio-pep-2 peptide conjugated [53,61,64,69,70,71,72]	Au-DOX@PO-ANG	Increased doxorubicin delivery across the BBB and prolonged survival
	ANG-LP-MSN-PTX	Improved BBB penetration and patient survival
	Angiopep-2-functionalized Saporin polymersomes	Enhanced antitumor effectiveness of Saporin while limiting systemic side effects
	Angiopep-2-conjugated PS loaded with DOX	Enhanced antitumor effectiveness of doxorubicin while limiting systemic side effects
	Angiopep-2-DOX with EGFR-targeting peptide (EP-1)	Improved selective targeting and therapeutic effectiveness
	KS487 cyclic peptide	Improved BBB penetration and targeted drug delivery
	ANG1005	
	GRN1005	
	DOX-TAT-Ang-LIP	
	PEGylated ANG2-functionalized zein nanoparticles	Improved drug delivery efficiency by reducing concern related to LRP1 receptor saturation
Apolipoprotein E-functionalized systems [53]	-	Improved penetration of BBB and drug delivery
Ferroptosis- and cell death-inducing therapies [56,57,69]	Angiopep-2-modified engineered exosomes with magnetic nanoparticles	Stimulates ferroptosis
	L-D-I/NPs	Stimulates ferroptosis
	Ti@FeAu-Ang	Hyperthermia-induced necrosis and decreased tumor volume
Photothermal and sonodynamic therapy [62,68]	ACHL nano-sensitizer (Ce6 + HCQ liposomes)	Improved sonodynamic antitumor effect while reducing toxicity to normal cells
	Angiopep-2-functionalized gold nanorods (GNRs)	Increased ROS production and photothermal killing of glioma cells
PAMAM dendrimer conjugated [54,55]	PAMAM dendrimer with doxorubicin (P4PAD)	Improved penetration of the BBB and drug delivery
Radiosensitizer-based therapy [65]	ch-K5(s-s)R8-An micellar system (Dbait + doxorubicin)	Improved radiotherapy responsiveness
	A2-P(MIs)25/TMZ nanoparticles	
	Dbait radiosensitizer	
LRP1 inhibitor [59]	RAP	Competitively inhibits LRP1 with potential anti-glioma effects

## Data Availability

No new data were created or analyzed in this study.

## Abbreviations

The following abbreviations are used in this manuscript:

BBB	Blood–brain barrier
CNS	Central nervous system
EGFRrs	Epidermal growth factor precursor repeats
EMT	Epithelial–mesenchymal transition
MMP	Matrix metalloproteinase
NRs	Nanorods
GBM	Glioblastoma
GRs	Goldrods
PAI-1	Plasminogen activator inhibitor-1
TME	Tumor microenvironment
TAMs	Tumor-associated macrophages
LDLR	Low-density lipoprotein receptor
LRP1	Low-density lipoprotein receptor-related protein 1
